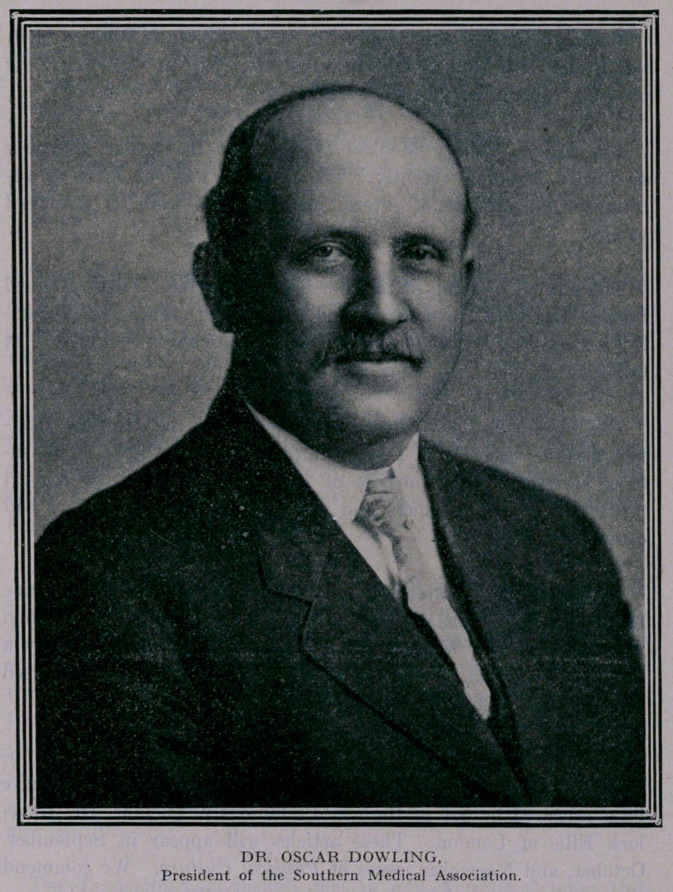# Dr. Oscar Dowling, President of the Southern Medical Association

**Published:** 1915-11

**Authors:** 


Dr. Oscar Dowling was born in Montgomery, Alabama, October 29, 1866; he is a son of Rev. Angus and Laura L. (Boswell) Dowling. He received his M. D. degree from Vanderbilt University in 1888. He has done postgraduate work in New York, Chicago and New Orleans polyclinics; New York Eye and Ear Infirmary, and has attended clinics in London, Berlin, Paris and Mexico City. He was county health officer of Henry county, Alabama, from 1892 to 1894.

Dr. Dowling is a specialist in the treatment of the eye, ear, nose and throat, and has occupied many important positions as oculist and aurist. He is a trustee of the American Medical Association; President of the Southern Medical Association; First Vice-President of the Southern Sociological Congress; one of the Vice-Presidents of the National Mouth Hygiene Association; a director of the American Public Health Association, and belongs to many prominent clubs. He has accomplished so much that it is impossible to recount all of his achievements in so brief an article.

When he was appointed President of the Louisiana State Board of Health, September, 1910, he opened a unique "Clean Up" campaign. He toured the State in a special train with health exhibit, inspecting the food and water supply of various communities, and also visited leading cities as far north as Chicago and as far west as the Pacific Coast.

By his untiring zeal and his earnest devotion to what he believed to be the duty of a State Health Officer, he has given the United States and foreign countries a new and higher ideal of the duties and responsibilities and power of a State Health Officer. Through his efforts Louisiana has a very rigid vital statistics law,

DR. OSCAR DOWLING,
President of the Southern Medical Association.

and the common drinking cup and common towel are practically unknown.

The South owes him much for his progressive ideas of sanitation and the Southern Medical Association honored itself in choosing him President.

## Figures and Tables

**Figure f1:**